# Prognosis of pregnancy-associated breast cancer: a meta-analysis

**DOI:** 10.1186/s12885-020-07248-8

**Published:** 2020-08-10

**Authors:** Chunchun Shao, Zhigang Yu, Juan Xiao, Liyuan Liu, Fanzhen Hong, Yuan Zhang, Hongying Jia

**Affiliations:** 1 Center of Evidence-based Medicine, Institute of Medical Sciences, The Second Hospital of Shandong University, Jinan, 250033 Shandong PR China; 2 Department of Breast Surgery, The Second Hospital of Shandong University, Jinan, 250033 Shandong PR China; 3 Department of Obstetrics, The Second Hospital of Shandong University, Jinan, 250033 Shandong PR China; 4 Clinical Epidemiology Unit, Qilu Hospital of Shandong University, Jinan, 250012 Shandong PR China; 5 Clinical Research Center of Shandong University, Jinan, 250012 Shandong PR China

**Keywords:** Pregnancy-associated breast cancer, Prognosis, Survival, Dose-response, Meta-analysis

## Abstract

**Background:**

Pregnancy-associated breast cancer (PABC) is defined as breast cancer that is diagnosed during pregnancy and/or the postpartum period. Definitions of the duration of the postpartum period have been controversial, and this variability may lead to diverse results regarding prognosis. Moreover, evidence on the dose-response association between the time from the last pregnancy to breast cancer diagnosis and overall mortality has not been synthesized.

**Methods:**

We systematically searched PubMed, Embase, and the Cochrane Library for observational studies on the prognosis of PABC published up to June 1, 2019. We estimated summary-adjusted hazard ratios (HRs) and the corresponding 95% confidence intervals (CIs). Subgroup analyses based on diagnosis time, PABC definition, geographic region, year of publication and estimation procedure for HR were performed. Additionally, dose-response analysis was conducted by using the variance weighted least-squares regression (VWLS) trend estimation.

**Results:**

A total of 54 articles (76 studies) were included in our study. PABC was associated with poor prognosis for overall survival (OS), disease-free survival (DFS) and cause-specific survival (CSS), and the pooled HRs with 95% CIs were 1.45 (1.30–1.63), 1.39 (1.25–1.54) and 1.40 (1.17–1.68), respectively. The corresponding reference category was non-PABC patients. According to subgroup analyses, the varied definition of PABC led to diverse results. The dose-response analysis indicated a nonlinear association between the time from the last delivery to breast cancer diagnosis and the HR of overall mortality (*P* < 0.001). Compared to nulliparous women, the mortality was almost 60% higher in women with PABC diagnosed at 12 months after the last delivery (HR = 1.59, 95% CI 1.30–1.82), and the mortality was not significantly different at 70 months after the last delivery (HR = 1.14, 95% CI 0.99–1.25). This finding suggests that the definition of PABC should be extended to include patients diagnosed up to approximately 6 years postpartum (70 months after the last delivery) to capture the increased risk.

**Conclusion:**

This meta-analysis suggests that PABC is associated with poor prognosis, and the definition of PABC should be extended to include patients diagnosed up to approximately 6 years postpartum.

## Background

Breast cancer is the second most common cancer worldwide and the most commonly occurring malignancy in women [1]. Due to the trend of delayed delivery, the number of women with breast cancer during a pregnancy or in the subsequent few years after a pregnancy is expected to increase [2]. Breast cancer occurring during pregnancy is a challenging clinical situation since the welfare of both the mother and the foetus must be considered in any treatment plan. Conventionally, pregnancy-associated breast cancer (PABC) is defined as breast cancer that is diagnosed during pregnancy or the postpartum period. Definitions of how many years after delivery breast cancer can be diagnosed under this definition have ranged from 0.5 to 5 years, and sometimes even longer [3, 4]. PABC is viewed as a clinically and biologically special type of breast cancer and only comprises 0.2–0.4% of all breast cancers [5, 6]. However, it is the most common cancer in pregnancy and is diagnosed in approximately 15 to 35 per 100,000 births, and the number of breast cancer cases diagnosed during pregnancy is less than after delivery [7–10].

Pregnancy itself may temporarily increase the risk of developing breast cancer, although it has a long-term protective effect on the development of breast cancer [11, 12]. However, whether PABC has a worse prognosis is currently controversial. A meta-analysis published in 2016 showed that the risk of death increased in women with PABC compared with women with non-PABC (pooled hazard ratio (HR), 1.57; 95% confidence interval (CI), 1.35–1.82) [13]. However, other recent studies found no significant difference in the prognosis of PABC and non-PABC [14–17]. Meanwhile, the specific definition of PABC has varied and this variability may lead to diverse results on the relationship among pregnancy, postpartum and breast cancer. Therefore, it is necessary to specify the definition of PABC by summarizing epidemiological evidence. This study was initiated to understand the prognosis of PABC and examine the dose-response relationship to provide quantitative evidence for defining PABC.

## Methods

### Search strategy

This meta-analysis was performed in accordance with the preferred reporting items for systematic reviews and meta-analyses (PRISMA) guidelines. We did our best to include studies published to date regarding the prognosis of PABC. Eligible studies were found by searching PubMed, Embase, and the Cochrane Library for relevant reports published before June 1, 2019. The keywords used for the search were (“pregnan*” OR “gestation*” OR “childbirth” OR “postpartum” OR “parity”) AND “breast” AND (“cancer” OR “neoplasia” OR “carcinoma”). The references lists of all retrieved articles and previous systematic reviews were manually searched.

### Inclusion and exclusion criteria

All eligible studies met the following criteria: (1) observational prognostic studies with a follow-up period longer than 6 months; (2) participants were diagnosed with breast cancer by clinical diagnosis and/or histologically; (3) the case group was diagnosed with PABC, and the control group was non-PABC or nulliparity; (4) the outcomes were in terms of overall survival (OS), disease-free survival (DFS) or cause-specific survival (CSS); and (5) the risk point estimate was reported as an HR with 95% CI, or the data were presented such that an HR with 95% CI could be calculated. The exclusion criteria were as follows: (1) duplicated or irrelevant articles; (2) reviews, letters, and case reports; (3) non-human studies; and (4) studies with inappropriate data for meta-analysis, such as incomplete or inconsistent data.

### Data extraction

Two reviewers extracted the data independently using a predefined data extraction form. Any disagreements were resolved by discussion. The extracted data included the first author, publication year, country, PABC definition, control definition, sample size, cancer type, stage or grade, age, matching criteria, adjusted variables, and adjusted HRs with 95% CIs.

### Assessment of study quality

The methodological quality of the studies was assessed by the Newcastle-Ottawa scale (NOS) [18]. A score of 0–9 was allocated to each study, with higher scores indicating higher quality.

### Meta-analysis and statistical analysis

We used adjusted HRs and 95% CIs, which are most appropriate for time-to-data events. If HRs were not reported, we estimated HRs from the raw data or Kaplan-Meier curves [19]. The I-square (I^2^) test was performed to assess the impact of study heterogeneity on the results of the meta-analysis. If severe heterogeneity was present at I^2^ > 50%, a random effects model was chosen; otherwise, a fixed effects model was used. Visual inspection of the funnel plot and Egger’s and Begg’s tests were performed to assess publication bias. Subgroup analyses were performed according to the diagnosis time, PABC definition, geographic region, year of publication and estimation procedure for HR.

Variance-weighted least squares regression (VWLS) model was used to evaluate the dose-response association between the time from the last pregnancy to breast cancer diagnosis and HR of overall mortality [20]. Restricted cubic splines were used to check the time from the last pregnancy as a continuous, nonlinear exposure, and the time was defined by the 5th, 35th, 65th and 95th percentiles of the distribution [21]. The time from the last pregnancy to breast cancer diagnosis reported in each study was converted to months. We used the average value of the lower and upper limits of each category. If the lowest category was open ended, the average value of the upper limit and 0 was used. If the highest category was open ended, the average value was defined as 1.5 times the lower limit. All statistical analyses were performed using STATA Version 13.0. *P* < 0.05 was considered significant.

## Results

### Search results and study characteristics

**Fig. 1:**
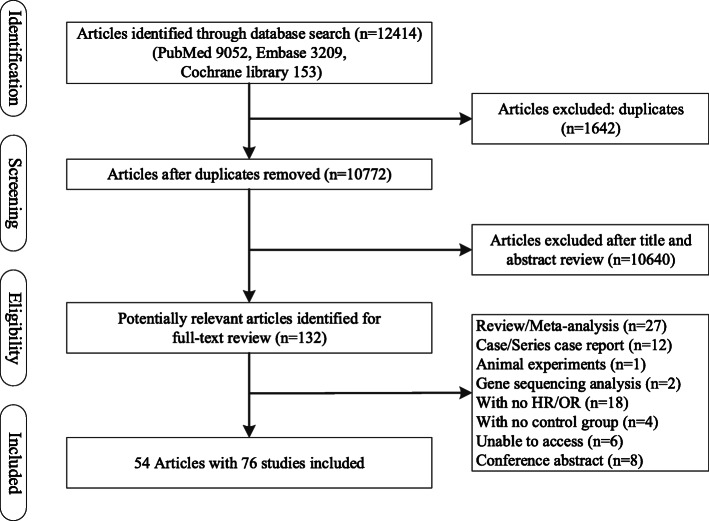
Schematic representation of the study selection process

We initially identified 12,414 articles and screened their titles and abstracts (Fig. 1). After duplicated and irrelevant articles were excluded, 54 articles with 76 studies met the inclusion criteria and were thus included in our meta-analysis. The quality of the studies was assessed based on the NOS and ranged from 6 to 9 (mean of 7.2). The characteristics of the studies are summarized in Table 1.

**Table 1:** Characteristics of the studies included in the meta-analysis

Study ID	Country	No. of PABC cases	No. of controls	PABC definition	Cancer stage or grade	Mean/median age of PABC	Follow-up years	Outcomes measured	HR estimate	HR	95% CI	NOS score	Matching criteria	Adjusting variable
Mausner, 1969 [22]	USA	73	647	Pregnancy & < 6 months postpartum	Stage I II III, Grade I II III	35	5	OS	indirect	1.36	1.07–1.73	7	–	–
Wallgren, 1977 [23]	Sweden	15	58	Pregnancy & < 12 months postpartum	Grade I II III	< 30	10	OS	indirect	1.35	0.71–2.58	7	–	–
Nugent, 1985 [24]	USA	19	155	Pregnancy	Stage I II III	32	5	OS	indirect	0.96	0.55–1.67	6	–	–
Tretli, 1988-Pregnancy [25]	Norway	20	40	Pregnancy	Stage I II III	33	4	OS	indirect	2.41	1.32–4.37	6	Diagnosed year, diagnosed age	–
Tretli, 1988-Postpartum [25]	Norway	15	40	Unspecified	Stage I II III	36	4	OS	indirect	1.47	0.66–3.27	6		–
Greene, 1988 [26]	USA	8	36	Pregnancy	NA	<35	14	OS	indirect	1.50	0.18–12.62	6	–	–
Petrek, 1991 [27]	USA	56	166	Pregnancy & < 12 months postpartum	NA	–	5	OS	paper	0.74	0.37–1.45	6	–	Node status
Zemlickis, 1992 [28]	Canada	102	269	Pregnancy & postpartum (unspecified)	Stage 0 I II III IV	33	25	CSS	indirect	1.25	0.93–1.69	8	Stage, age at diagnosis	–
Ishida, 1992 [29]	Japan	192	191	Pregnancy & < 24 months postpartum	Stage 0 Tis I II III IV	32	10	OS	indirect	2.00	1.27–3.16	6	–	
Guinee, 1994-Pregnancy [30]	USA	26	139	Pregnancy	NA	28(20–29)	10	OS	paper	2.83	1.24–6.45	8	–	Tumour size, number of positive axillary nodes
Guinee, 1994-Postpartum [30]	USA	40	139	< 12 Months postpartum	NA	28(20–29)	10	OS	paper	1.88	0.88–3.98	8	–	
Von Schoultz, 1995 [31]	Sweden	173	1740	Pregnancy & < 60 months postpartum	NA	< 50	7	DFS	paper	1.02	0.72–1.43	9	–	Age, nodal status, tumour size, ER status
Ezzat, 1996-OS [32]	Saudi Arabia	28	84	Pregnancy	Stage I II III	20–45	7	OS	paper	0.90	0.6–1.3	6	Year of diagnosis, date of beginning	–
Ezzat, 1996-DFS [32]	Saudi Arabia	28	84	Pregnancy	Stage I II III	20–45	7	DFS	paper	1.10	0.8–1.5	6		–
Anderson, 1996-OS [33]	USA	22	205	Pregnancy & < 12 months postpartum	Stage 0 I II IIIa	< 30	10	OS	paper	2.40	1.28–4.50	8	–	Stage, axillary LN involvement, adjuvant CT, tumour size
Anderson, 1996-DFS [33]	USA	22	205		Stage 0 I II IIIa	< 30	10	DFS	indirect	3.19	1.20–8.49	8	–	
Bonnier, 1997-OS [34]	France	154	308	Pregnancy & < 6 months postpartum	Grade I II III	33.9(23.2–46.4)	5	OS	paper	1.46	0.72–2.96	6	–	Clinical tumour size, microscopic lymph-node involvement, inflammatory cancer, age
Bonnier, 1997-DFS [34]	France	154	308		Grade I II III		5	DFS	paper	1.48	1.00–2.19	6	–	
Olson, 1998 [35]	USA	146	–	–	NA	< 45	15	OS	paper	–	–	7	–	Age, tumour size, lymph nodes, ER status, histology
Reeves, 2000 [36]	UK	–	–	–	Stage I II III IV	< 60	> 10	OS	paper	–	–	9	–	Age at diagnosis, year of diagnosis, hospital, weight in kg
Ibrahim, 2000 [37]	Saudi Arabia	72	216	Pregnancy	Stage I II III IV, Grade I II III	34	10	OS	indirect	0.94	0.62–1.44	6	Age, stage, year of diagnosis	–
Daling, 2002 [38]	USA	83	309	< 24 Months postpartum	Stage I II III IV	< 45	5	OS	indirect	2.30	1.4–3.9	9	–	Age, diagnosis year
Aziz, 2003 [39]	Pakistan	24	48	Pregnancy & < 12 months postpartum	NA	32(20–45)	7	OS	indirect	1.67	0.82–3.41	6	Age, tumour grade, tumour size, axillary lymph node status	–
Siegelmann-Danieli, 2003-OS [40]	Israel	22	192	Pregnancy & < 12 months postpartum	NA	33(25–27)	5	OS	indirect	3.39	0.58–19.81	6	–	–
Siegelmann-Danieli, 2003-DFS [40]	Israel	20	181		NA	33(25–28)	5	DFS	indirect	4.81	1.46–15.9	6	–	–
Bladstrom, 2003 [41]	Sweden	94	14,599	Pregnancy	NA	≤45	5	OS	paper	2.40	2.0–2.9	9	–	Age, time of diagnosis, time period interaction, number of children, age at first child’s birth
Bladstrom, 2003(2) [41]	Sweden	94	14,599	Pregnancy	NA	≤45	10	OS	paper	1.20	0.9–1.7	9	–	
Whiteman, 2004 [42]	USA	59	355	< 12 Months postpartum	NA	20–45	15	OS	paper	1.51	1.02–2.23	9	–	Surgery, radiation therapy, race, oral contraceptive use, education, BMI, stage history of breast disease
Rodriguez, 2008 [43]	USA	797	4177	Pregnancy & < 12 months postpartum	Stage I II III IV	< 55	13	OS	paper	1.14	1.00–1.29	9	–	Race, tumour size, AJCC stage, surgery, hormone receptor
Stensheim, 2009-Pregnancy [44]	Norway	59	13,106	Pregnancy	NA	< 50	5	CSS	paper	1.23	0.82–1.81	7	–	Age, diagnostic period, initial extent of disease
Stensheim, 2009-Postpartum [44]	Norway	46	13,106	< 6 Months postpartum	NA	< 50	5	CSS	paper	1.95	1.36–2.78	7	–	
Beadle, 2009-OS [45]	USA	104	564	Pregnancy & < 12 months postpartum	Stage I II III	≤35	10	OS	indirect	1.24	0.87–1.79	6	–	–
Beadle, 2009-DFS (distant metastasis) [45]	USA	104	564	Pregnancy & < 12 months postpartum	Stage I II III	≤35	10	DFS	indirect	1.35	0.98–1.85	6	–	–
Beadle, 2009-DFS (locoregional recurrence) [45]	USA	104	564		Stage I II III	≤35	10	DFS	indirect	1.44	0.78–2.66	6	–	–
Halaska, 2009-OS [46]	Greece	32	32	Pregnancy & < 12 months postpartum	Grade I II III	< 45	10	OS	indirect	1.42	0.58–3.48	6	Age at diagnosis, tumour size, axillary lymph node status, presence or absence of metastatic deposits	–
Halaska, 2009-DFS [46]	Greece	32	32		Grade I II III	< 45	10	DFS	indirect	1.82	0.82–4.05	6		–
Largillier, 2009-OS [47]	France	105	788	Pregnancy & < 12 months postpartum	Grade I II III	<35	10	OS	paper	1.51	1.05–2.20	7	–	–
Largillier, 2009-DFS [47]	France	105	788		Grade I II III	<35	10	DFS	paper	1.25	0.90–1.74	7	–	–
Phillips, 2009 [48]	Multicentre	676	–	–	NA	–	10	OS	paper	–	–	8	–	Study centre, education, BMI, time since last full-term pregnancy, age at diagnosis
Moreira, 2010 [49]	Brazil	87	252	Pregnancy & < 12 months postpartum	NA	≤ 45	10	OS	paper	1.52	1.10–2.10	7	Registration institution, age, registration year	–
Johansson, 2011 [50]	Sweden	1110	14,611	Pregnancy & < 24 months postpartum	NA	15–44	15	OS	paper	1.51	1.36–1.68	7	–	Age, calendar time, education
Murphy, 2012 [51]	USA	99	186	Pregnancy & < 12 months postpartum	Grade 0 I II III	35(24–48)	18	OS	paper	0.59	0.29–1.17	7	Age, year of diagnosis	Tumour grade, ER status, LN involvement
Azim, 2012-OS [52]	Italy	65	130	Pregnancy	NA	< 50	6	OS	paper	1.70	0.80–3.90	7	Age, year of surgery, pathological tumour size, pathological nodal status	pN, neoadjuvant chemotherapy, ER
Azim, 2012-DFS [52]	Italy	65	130	Pregnancy	NA	< 50	6	DFS	paper	2.30	1.30–4.20	7		Age, pT, pN, neoadjuvant chemotherapy, Ki-67, HER2, perivascular invasion
Ali, 2012-OS [53]	USA	40	40	Pregnancy & < 12 months postpartum	Stage I II III IV	33(24–42)	16	OS	indirect	2.15	1.13–4.09	7	–	Age and stage-matched
Ali, 2012-DFS [53]	USA	40	40		Stage I II III IV	33(24–42)	16	DFS	indirect	2.00	1.12–3.59	7	–	
Amant, 2013-OS [54]	Belgium	311	865	Pregnancy	Stage I II III, Grade I II III	33(31–36)	5	OS	paper	1.19	0.73–1.93	8	–	Age at diagnosis, stage, grading, histologic tumour type, ER/PR status, HER2, chemotherapy
Amant, 2013-DFS [54]	Belgium	311	865	Pregnancy	Stage I II III, Grade I II III	33(31–36)	5	DFS	paper	1.34	0.93–1.91	8	–	
Litton, 2013-OS [55]	USA	75	150	Pregnancy	Stage I II III	24–45	5	OS	paper	1.87	1.04–3.36	7	Age at diagnosis, stage at diagnosis, year of diagnosis	Age at diagnosis, year of diagnosis, clinical cancer stage, tumour nuclear grade
Litton, 2013-DFS [55]	USA	75	150	Pregnancy	Stage I II III	24–45	5	DFS	paper	2.09	1.19–3.67	7		
Valentini, 2013 [56]	USA	75	269	Pregnancy & < 12 months postpartum	NA	32.5(20–45)	15	OS	paper	0.79	0.25–2.44	7	–	Age at diagnosis, tumour size, lymph node status, ER status, use of chemotherapy, oophorectomy
Dimitrakakis, 2013 [57]	Greece	39	39	Pregnancy & < 12 months postpartum	Stage I II III IV, Grade I II III	34.3 ± 5.0	5	OS	paper	9.28	2.94–29.27	6	Stage, age, year of diagnosis	Stage, ER status, grade, age at diagnosis
Calliha, 2013-OS [58]	USA	76	86	Pregnancy & < 60 months postpartum	Stage 0 I II III IV, Grade I II III	≤45	5	OS	paper	2.65	1.09–6.42	6	–	Tumour biological subtype, clinical stage, year of diagnosis
Calliha, 2013-DFS [58]	USA	74	84	Pregnancy & < 60 months postpartum	Stage 0 I II III IV, Grade I II III	≤45	5	DFS	paper	2.80	1.12–6.57	6	–	Tumour biological subtype, clinical stage, year of diagnosis, local recurrence
Bell, 2013-OS [59]	Australia	13	377	Pregnancy & < 12 months postpartum	NA	< 48	5	OS	paper	2.50	0.5–11.7	6	–	–
Bell, 2013-DFS [59]	Australia	13	377	Pregnancy & < 12 months postpartum	NA	< 48	5	DFS	paper	0.90	0.2–4.4	6	–	–
Moller, 2013 [60]	UK	–	–	–	Stage I II III IV	10–54	10	OS	paper	–	–	7	–	Age, stage
Framarino-dei-Malatesta, 2014 [61]	Italy	22	45	Pregnancy	NA	37.2 ± 3.2	10	OS	indirect	0.96	0.29–3.21	6	Age	–
Madaras, 2014 [62]	Hungary	31	31	Pregnancy & < 12 months postpartum	–	34	10	OS	indirect	5.76	2.09–15.98	7	Age, year of first breast cancer diagnosis	–
Nagatsuma, 2014 [63]	Japan	–	–	–	Stage 0 I II III IV, Grade I II III	26–44	10	OS	paper	–	–	7	–	Age at diagnosis, AJCC clinical stage, histological tumour grade, oestrogen and progesterone receptor status, HER2 status
Strasser-Weippl, 2014 [64]	China	109	1274	Pregnancy & < 60 months postpartum	Grade I II III	< 45	5	DFS	paper	1.62	1.04–2.54	8	–	Age, oestrogen receptor, progesterone receptor, HER2 status, disease stage
Genin, 2015-OS [65]	France	87	174	Pregnancy & < 12 months postpartum	Grade I II III	35(27–40)	10	OS	indirect	1.09	0.79–1.52	7	Age, year of diagnosis	–
Genin, 2015-DFS [65]	France	87	174	Pregnancy & < 12 months postpartum	Grade I II III	35(27–40)	10	DFS	paper	1.87	1.05–3.33	7	Age, year of diagnosis	Age, ER, HR status, tumour stage, HER2 status, Ki-67 rate
Iqbal, 2017 [14]	Canada	501	5832	Pregnancy & < 21 months postpartum	Stage I II III IV	20–45	5	OS	paper	1.11	0.86–1.45	9	–	Year of diagnosis, age, tumour size, nodal status, oestrogen receptor status, progesterone receptor status, chemotherapy, radiotherapy, et al
Kim, 2017 [66]	Korea	344	668	Pregnancy & < 12 months postpartum	Stage 0 I II III IV, Grade I II III	20–45	10	OS	indirect	1.85	1.28–2.67	8	Operation period, age, initial stage	–
Bae, 2018(1) [67]	Korea	40	2770	Pregnancy & < 12 months postpartum	Stage 0 I II III	33.5 (27–40)	5	CSS	paper	4.00	1.20–12.90	8	–	Age, stage, chemotherapy
Bae, 2018(2) [68]	Korea	411	83,381	Pregnancy & < 12 months postpartum	Stage 0 I II III IV	20–49	15	OS	paper	1.03	0.74–1.42	9	–	Age at diagnosis, stage, high versus low/intermediate, luminal subtype, HER2 subtype, et al
Boudy, 2018-DFS [16]	France	49	51	Pregnancy	Grade I II III	< 46	5	DFS	indirect	1.19	0.75–1.91	8	Propensity score	–
Boudy, 2018-CSS [16]	France	49	51	Pregnancy	Grade I II III	< 46	5	CSS	indirect	1.06	0.65–1.72	8		–
Johansson, 2018 [2]	Sweden	778	1661	Pregnancy & < 24 months postpartum	Stage 0 I II III IV	15–44	10	OS	indirect	0.90	0.55–1.40	9	–	Age, period, education, region, tumour characteristics, pathologic T stage, N stage, ER/PR
Chuang, 2018 [69]	China (Taiwan)	–	–	–	Stage I II III	20–50	> 10	OS	paper	–	–	9	–	Age and year of diagnosis, stage, tumour size, positive lymph nodes, histological grading, treatments
Ploquin, 2018-OS [15]	France	111	253	Pregnancy	Stage 0 I II III IV	22–46	5	OS	paper	1.10	0.67–1.79	8	Age, clinical T stage, hormone receptor	Clinical nodal status, age
Ploquin, 2018-DFS [15]	France	111	253	Pregnancy	Stage 0 I II III IV	22–46	5	DFS	paper	1.15	0.78–1.68	8		
Suleman, 2019-OS [70]	Saudi Arabia	110	114	Pregnancy	Stage I II III IV	20–48	> 10	OS	indirect	2.58	1.26–5.26	7	Diagnosed year	–
Suleman, 2019-DFS [70]	Saudi Arabia	110	114	Pregnancy	Stage I II III IV	20–48	> 10	DFS	indirect	1.18	0.70–1.97	7		–
Choi, 2019 [17]	Korea	63	3804	Pregnancy & < 12 months postpartum	NA	< 50	10	OS	paper	1.52	0.82–2.83	8	–	Histologic type, stage, ER, PR, age at diagnosis, Charlson comorbidity index

*BMI* Body mass index, *ER* Oestrogen receptor, *PR* Progesterone receptor, *HER-2* Human epidermal growth factor receptor-2

### Overall survival (OS)

**Fig. 2:**
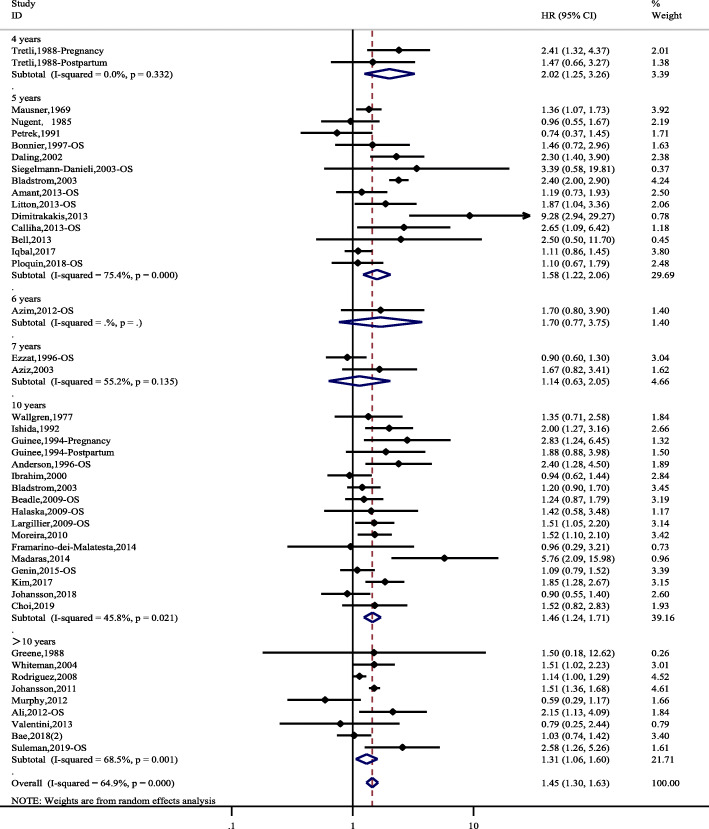
Hazard ratios and 95% CIs of studies included in the meta-analysis of OS

Forty-five studies comprising 6602 PABC patients and a total of 157,657 individuals were identified for the meta-analysis of OS. There was an overall increased risk of death for PABC patients compared to controls, with a pooled hazard ratio of 1.45 (95% CI 1.30–1.63). There was significant heterogeneity (*I*^*2*^ = 64.9, *P*<0.001). The subgroup analysis according to different follow-up durations (4 years, 5 years, 6 years, 7 years, 10 years and > 10 years) had similar results to the overall analysis (Fig. 2). However, the 6-year and 7-year OS, with few studies, showed nonsignificant results.

### Disease-free survival (DFS)

**Fig. 3:**
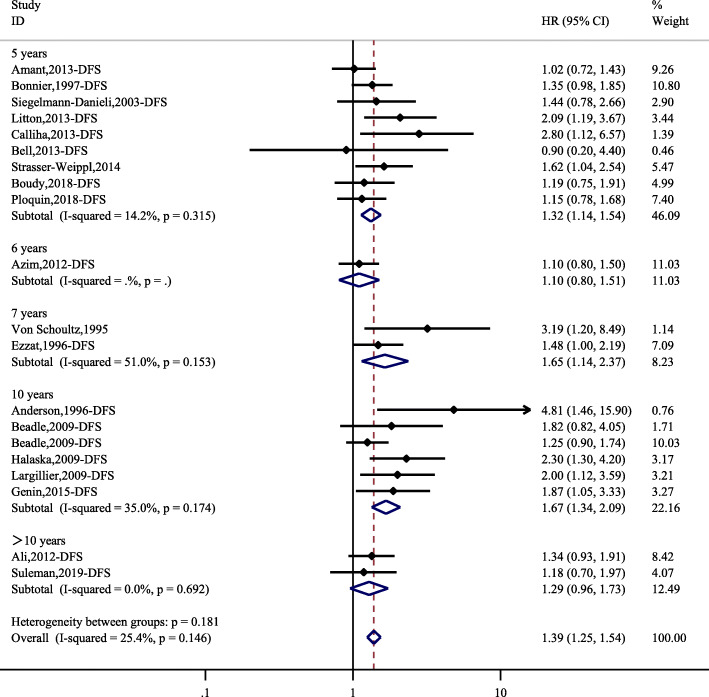
Hazard ratios and 95% CIs of studies included in the meta-analysis of DFS

Twenty studies comprising 1786 PABC patients and a total of 9762 individuals were identified for the meta-analysis of DFS. The overall HR was 1.39 (95% CI, 1.25–1.54). There was no significant heterogeneity (*I*^*2*^ = 24.5, *P* = 0.146). The subgroup analysis according to different follow-up durations (5 years, 6 years, 10 years and > 10 years) had similar results as the overall analysis (Fig. 3). However, the 7-year DFS, with only 2 studies, showed nonsignificant results.

### Cause-specific survival (CSS)

**Fig. 4:**
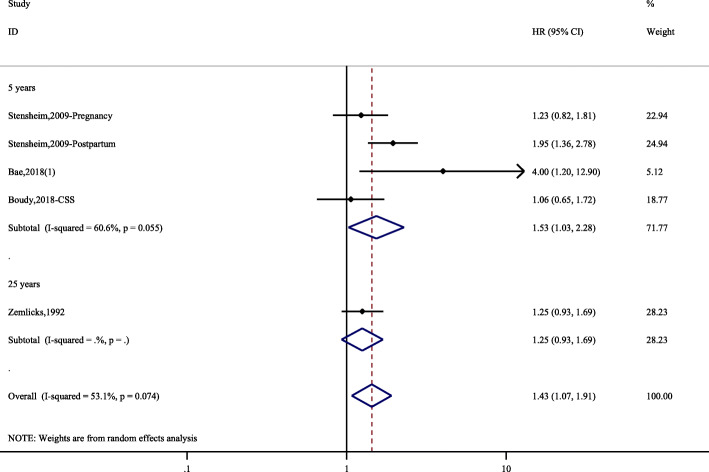
Hazard ratios and 95% CIs of studies included in the meta-analysis of CSS

Only 6 studies provided information on CSS with 296 PABC patients and a total of 29,598 individuals. The overall HR was 1.40 (95% CI, 1.17–1.68). There was no significant heterogeneity (*I*^*2*^ = 53.1, *P* = 0.074). The subgroup analysis (5-year CSS) had similar results as the overall analysis (Fig. 4).

### Subgroup analyses

Several factors that may have induced differences in outcomes were investigated with subgroup analyses, including diagnosis time, PABC definition, geographic region, year of publication and estimation procedure for HR. The results consistently showed worse prognoses in women with PABC than in those with non-PABC, except for the subgroup based on PABC definition and year of publication (Table 2). It is worth noticing that the specific definition has varied and this variability led to diverse results. Studies published during the years 2000–2010 and 2011–2019 had a clear trend of poor prognoses, which was less apparent in those published before 2000. The pooled HR of DFS based on studies published before 2000 was 1.27 (95% CI, 0.97–1.72).

**Table 2:** Subgroup analyses

Subgroups			No. of Articles (No. of Studies)	HR (95% CI)	Heterogeneity Test	
					*I*^*2*^ *(%)*	*P*-value
All studies included			54 (76)	–	–	–
Diagnosed time	During pregnancy	OS	13 (14)	1.46(1.12–1.90)	73.6	< 0.001
		DFS	7 (7)	1.30(1.11–1.53)	26.3	0.228
	During postpartum period	OS	13(13)	1.97(1.67–2.33)	49.0	0.023
		DFS	2(2)	1.86(1.17–2.93)	0.0	0.740
PABC definition	Pregnancy & < 6 months postpartum	OS	2(2)	1.37(1.09–1.72)	0.0	0.852
	Pregnancy & < 12 months postpartum	OS	20(20)	1.44(1.20–1.72)	60.7	< 0.001
		DFS	8(9)	1.52(1.27–1.81)	17.4	0.288
	Pregnancy & < 24 months postpartum	OS	3(3)	1.42(1.01–2.01)	67.4	0.047
	Pregnancy & < 60 months postpartum	OS	3(3)	1.48(0.90–2.44)	65.2	0.057
Geographic region	Europe	OS	15(17)	1.53(1.26–1.86)	71.1	< 0.001
		DFS	9(9)	1.32(1.15–1.52)	8.7	0.363
	North America	OS	16(17)	1.38 (1.17–1.63)	53.2	0.005
		DFS	5(6)	1.68(1.35–2.08)	15.5	0.315
	Asia	OS	9(9)	1.42(1.09–1.85)	60.0	0.010
	Others	OS	2(2)	1.55(1.13–2.13)	0.0	0.544
Year of publication	Before 2000	OS	11(13)	1.46(1.18–1.82)	45.4	0.038
		DFS	3(3)	1.27(0.97–1.72)	50.7	0.107
	2000–2010	OS	11(12)	1.48(1.19–1.85)	79.0	< 0.001
		DFS	4(5)	1.40(1.14–1.71)	20.5	0.284
	2011–2019	OS	20(20)	1.43(1.20–1.72)	62.7	< 0.001
		DFS	11(11)	1.50(1.29–1.76)	11.5	0.334
HR estimate	Paper report	OS	24(25)	1.42(1.22–1.65)	73.1	< 0.001
		DFS	12(12)	1.35(1.19–1.53)	29.1	0.160
	Indirect	OS	19(20)	1.43(1.28–1.60)	47.4	0.010
		DFS	7(8)	1.48(1.22–1.79)	24.7	0.232

### Dose-response association between the time from the last pregnancy to breast cancer diagnosis and HR of overall mortality

**Fig. 5:**
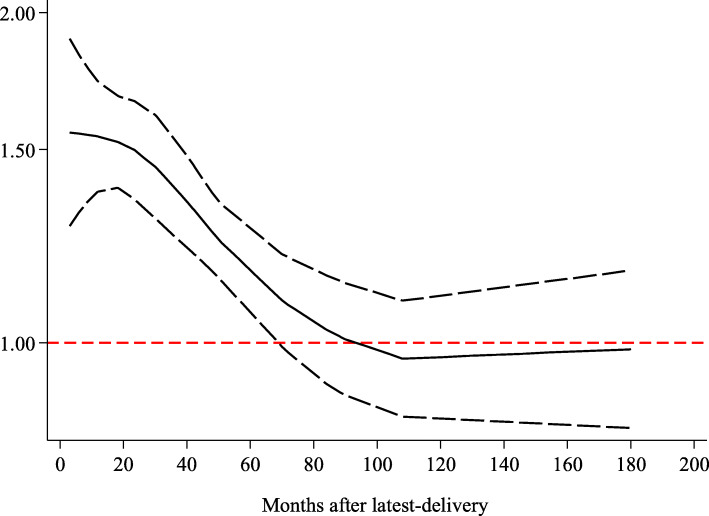
Dose-response relation between the time from the last delivery to breast cancer diagnosis and the HR of overall mortality

As the meta-analysis included studies reporting the HRs with their 95% CIs of overall mortality relating to three or more categories of time since the last pregnancy, all the studies were eligible to be included in the dose-response analysis. A total of ten studies were included in the dose-response meta-analysis, and nulliparous women were taken as the corresponding reference category (Table 3). The analysis of departure from linearity indeed indicated a nonlinear association between the time from the last delivery to breast cancer diagnosis and the hazard ratio of PABC overall mortality (*P* < 0.001). The nonlinear spline showed a decreasing trend. Compared to nulliparous women, the mortality was almost 60% higher in women with PABC diagnosed at 12 months after the last delivery (HR = 1.59, 95% CI 1.30–1.82), and the mortality was not significantly different at 70 months after the last delivery (HR = 1.14, 95% CI 0.99–1.25) (Fig. 5). These results showed a higher risk of death than that in nulliparous patients, suggesting that the definition of PABC should be extended to include patients diagnosed up to approximately 6 years postpartum (70 months since the last delivery) to capture the increased risk.

**Table 3:** Characteristics of the studies included in the dose-analysis meta-analysis

Study ID	Time point of breast cancer diagnosis	Time after last delivery (months)	No. of participants	Adjusted HR^a^	95% CI
Guinee, 1994 [30]	Postpartum 1–12 m	1–12	40	1.88	0.88–3.98
	Postpartum 13–48 m	13–48	51	1.09	0.54–2.19
	Postpartum ≥49 m	≥49	35	0.54	0.19–1.55
Olson, 1998 [35]	Postpartum < 24 m	0–24	42	3.1	1.8–5.4
	Postpartum ≥24 m	≥24	352	1.3	0.9–2.0
Reeves, 2000 [36]	Postpartum < 60 m	0–60	67	1.56	1.01–2.42
	Postpartum 60–108 m	60–108	80	0.88	0.58–1.32
	Postpartum > 120 m	> 120	525	0.99	0.77–1.27
Daling, 2002 [38]	Postpartum < 24 m	0–24	83	2.3	1.5–3.4
	Postpartum 24–60 m	24–70	120	1.5	1.0–2.1
	Postpartum > 60 m	> 70	661	1.2	0.9–1.6
Whiteman, 2004 [42]	Postpartum ≤12 m	0–12	59	1.51	1.02–2.23
	Postpartum 13–48 m	13–48	213	1.25	0.95–1.64
	Postpartum > 48 m	> 48	1470	1.06	0.86–1.31
Phillips, 2009 [48]	Postpartum < 24 m	0–24	133	2.75	1.98–3.83
	Postpartum 24–60 m	24–60	231	2.2	1.65–2.94
	Postpartum ≥72 m	≥72	2067	0.98	0.79–1.22
Calliha, 2013 [58]	Postpartum < 60 m	0–60	86	2.65	1.09–6.42
	Postpartum ≥60 m	≥60	172	1.52	0.71–3.28
Nagatsuma, 2014 [63]	Postpartum ≤24 m	0–24	37	2.19	1.05–4.56
	Postpartum 36–60 m	36–60	59	1.49	0.79–2.83
	Postpartum > 60 m	> 60	181	0.81	0.46–1.43
Johansson, 2018 [2]	Postpartum 0–6 m	0–6	41	1.16	0.64–2.14
	Postpartum 6–12 m	6–12	84	1.3	0.83–2.03
	Postpartum 12–24 m	12–24	194	1.01	0.70–1.46
	Postpartum 24–60 m	24–60	629	1.22	0.96–1.55
	Postpartum 60–120 m	60–120	1106	1.08	0.87–1.53
	Postpartum > 120 m	> 120	1623	0.98	0.78–1.22
Chuang, 2018 [69]	Postpartum 0–12 m	0–12	347	1.29	0.96–1.74
	Postpartum 13–24 m	13–24	410	1.27	0.95–1.70
	Postpartum 25–60 m	25–60	1583	1.06	0.88–1.27

^a^Corresponding reference category: nulliparous

### Publication Bias

**Fig. 6:**
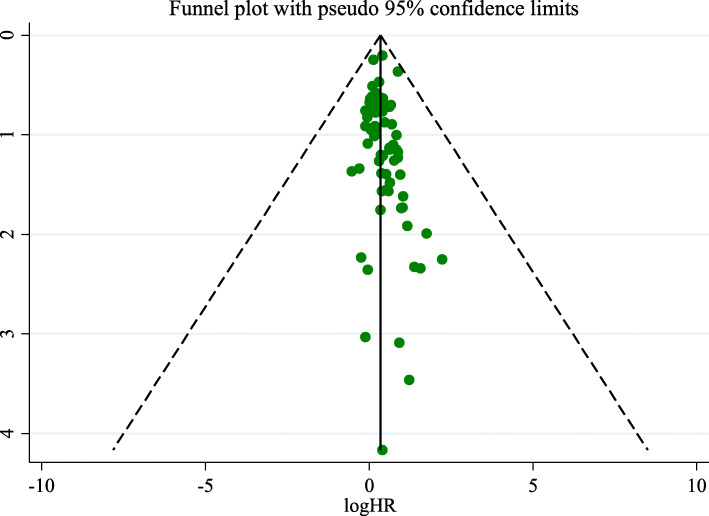
Funnel plot to explore the presence of publication bias

As shown in Fig. 6, each point represents an independent study of the indicated association, and a visual inspection of the funnel plot did not suggest evidence of publication bias among the articles (Egger’s test, *P* = 0.451; Begg’s test, *P* = 0.077).

## Discussion

We reviewed and meta-analyzed the existing scientific literature on the prognosis of PABC to draw a powerful conclusion that PABC is associated with a poor prognosis. Our results are consistent with those of the previous meta-analysis conducted in 2016 [13]. However, the negative effect on OS and DFS appears to be less pronounced in our study overall than in the previous meta-analysis. This is the largest and latest meta-analysis in this field. It included a larger number of participants, thus reducing the small-study effect to a great degree. The studies included in our meta-analysis were of relatively high quality. The mean Newcastle-Ottawa score of the studies was 7.2.

There are two explanations that may account for the results. On the one hand, mammary gland involution following pregnancy has been suggested to explain the poor prognosis [71]. Breast degeneration is the process of tissue remodelling, until wound healing, inflammatory bowel disease and immune infiltration reach a state indistinguishable from the non-productive breast [72, 73], which supposedly promotes tumour progression. On the other hand, pregnancy and breastfeeding lead to less timely detection and clinical examination. The delayed diagnosis allows more time for tumour growth, increasing the metastatic potential of the disease [52, 74]. Pregnancy also makes the treatment strategy more conservative to ensure the safety of the foetus [10, 75]. However, the exact reasons for the poor prognosis of PABC need to be explored in the future.

To the best of our knowledge, this is the first dose-response meta-analysis providing comprehensive insights into the association between the time from the last pregnancy to breast cancer diagnosis and the overall mortality of PABC. The scientific value of dose-response meta-analyses is higher than meta-analyses with exposure classified as two categories [20, 76]. Through the variance weighted least-squares regression with a random effects model, we found a nonlinear direct association between the time from the last pregnancy to breast cancer diagnosis and overall mortality. Compared with nulliparous women, the mortality was almost 60% higher in women with PABC diagnosed at 12 months after the last delivery, and the mortality had no significant difference at 70 months after the last delivery. We propose that the definition of PABC should include patients diagnosed up to at least 6 years postpartum to better delineate the increased risk imparted by a postpartum diagnosis. These findings also provide valuable insights into further research. Callihan’s cohort demonstrated that breast cancer patients diagnosed within 5 years postpartum have a significantly higher risk of metastasis and mortality than nulliparous patients [58]. Compared to that cohort, our dose-response meta-analysis provides a higher quality of evidence to expand the definition of PABC. Understanding the differences between breast cancers diagnosed during different times postpartum would better permit the translation of informative data from basic science and epidemiologic studies into the clinical care and treatment of breast cancer in young women.

The present meta-analysis has the following limitations that must be taken into account. First, if HRs and 95% CIs were not directly reported in the included studies, we estimated HRs from the crude data or Kaplan-Meier curves. This may cause bias without adjustment. However, we performed subgroup analysis based on the estimation procedure for HR. This analysis consistently showed a worse prognosis for women with PABC than for those with non-PABC. Second, the meta-analysis was based on data from observational studies; although most of the included studies adjusted for several relevant confounders (including age, year of diagnosis, tumour stage, axillary lymph node status, oestrogen receptor, hormonal receptor status, HER2 status, family history, etc.), residual confounding by other potential factors cannot be ruled out. Third, high between-study heterogeneity is another limitation of the current meta-analysis. This was likely due to significant differences in the sample sizes, definitions of PABC and/or treatment interventions. Last, the language of the studies was limited to English, which may result in potential language bias.

## Conclusions

In summary, this meta-analysis suggests that PABC is associated with a poor prognosis for OS, DFS and CSS compared to non-PABC cases. The definition of PABC should be extended to include patients diagnosed up to approximately 6 years postpartum to capture the increased risk of death. Further long-term prospective cohort studies with larger sample sizes should be conducted to validate this article’s findings.

## Data Availability

Not applicable.

## Abbreviations

PABC: Pregnancy-associated breast cancer
HR: Hazard ratio
CI: Confidence interval
VWLS: Variance weighted least-squares regression
OS: Overall survival
DFS: Disease-free survival
CSS: Cause-specific survival
PRISMA: Preferred reporting items for systematic reviews and meta-analyses
NOS: Newcastle-Ottawa Scale
BMI: Body mass index
ER: Oestrogen receptor
PR: Progesterone receptor
HER-2: Human epidermal growth factor receptor-2
